# Expression of mRNA vascular endothelial growth factor in hypospadias patients

**DOI:** 10.1186/s12894-021-00930-4

**Published:** 2021-11-27

**Authors:** Prahara Yuri, Gunadi, Rahmadani P. Lestari, Firly P. Fardilla, Ishandono Dachlan

**Affiliations:** 1 Division of Urology, Department of Surgery, Faculty of Medicine, Public Health and Nursing, Universitas Gadjah Mada/Dr. Sardjito Hospital, Jl. Kesehatan No.1, Yogyakarta, 55281 Indonesia; 2 Pediatric Surgery Division, Department of Surgery, Faculty of Medicine, Public Health and Nursing, Universitas Gadjah Mada/Dr. Sardjito Hospital, Yogyakarta, Indonesia; 3 Division of Plastic, Aesthetic and Reconstructive Surgery, Department of Surgery, Faculty of Medicine, Public Health and Nursing, Universitas Gadjah Mada/Dr. Sardjito Hospital, Yogyakarta, Indonesia

**Keywords:** Hypospadias, *VEGF*, Dartos tissue, Elasticity, Chordee, Penile curvature

## Abstract

**Background:**

Hypospadias is a relatively common genital anomaly in humans, usually followed by inelastic dartos that causes penile chordee. Vascular endothelial growth factor (*VEGF*) is strongly linked to the viscoelasticity of tissues and their elastic phase. This study aimed to evaluate *VEGF* expressions in (1) fascia dartos between hypospadias and controls and (2) chordee severity.

**Methods:**

This prospective cohort study involved 65 specimens from patients with hypospadias and ten specimens from controls. The samples were analyzed by quantitative real-time polymerase chain reaction (qPCR) for *VEGF* expression.

**Results:**

The expressions of *VEGF* were not different between proximal and distal hypospadias patients and controls (fold change: distal − 0.25; fold change: proximal − 0.2; *p* = 0.664). The scaled expressions related to chordee severity were mild − 0.1; moderate 0.1; severe − 0.25 (*p* = 0.660).

**Conclusions:**

*VEGF* expressions might not affect the severity of hypospadias and chordee, implying the pathogenesis is complex involving many growth factors. Further study with a larger sample size is necessary to clarify and confirm our findings.

## Introduction

Hypospadias, a relatively common human genital abnormality, is a urethral opening disorder that is not positioned correctly at the tip of the penis. Hypospadias is the second most common congenital anomaly in boys and the most common type of penis deformity [1, 2]. Molecular processes are needed in the occurrence of hypospadias, but the relationships and functions of these two elements are still unknown [3]. The exact cause of hypospadias has been identified in about 20% of cases, especially in the most challenging conditions [1].

Hypospadias may be related to an abnormality of the dartos fascia. However, this hypothesis is not supported by solid evidence from histopathology. There are contrasting studies of histopathological aspects of connective tissue in patients with hypospadias. The dartos fascia of hypospadias had abnormal tissue that gave it the thick and inelastic character [4, 5]. Inelastic dartos fascia tissue in patients diagnosed with hypospadias is a pathological tissue [4, 6, 7].

*VEGF* regulates elastic modulus and increases matrix stiffness in cardiomyocytes [8]. It is strongly associated with tissue viscoelasticity and the elastic cervix phase shortening [9]. *In addition, VEGF* plays a pivotal part in endothelial cells' proliferation, migration, and differentiation [10]. *VEGF* induces α1β1 and α2β1 integrins expression in microvascular endothelial cells [11], endothelial cell migration, and proliferation [12, 13]. *VEGF* is not retained intra-cellularly but attaches to the cell surface or extracellular membrane (ECM) and other matrix metalloproteinases (MMPs) [14]. However, the role of *VEGF* on dartos tissue remains unknown. Hence, this study aimed to investigate the impact of *VEGF* modulated dartos on tissue elasticity.

## Methods

### Patients

A prospective cohort study was performed in 65 patients who underwent repair of hypospadias with chordee without DSD, and ten patients underwent circumcision as controls between 2018 and 2020. The samples were then divided into three groups: distal hypospadias, proximal hypospadias, and controls. We used periurethral dartos tissues collected during excision of chordee. The Ethical Committee of our university approved this study(KE/0287/03/2020), and written informed consent to participate in this study was obtained from all the parents.

### qPCR analysis

All dartos tissue of each patient was placed in a single tube. All samples were homogenized. Total RNA was extracted using Hybrid-R™ Isolation Kit, and cDNA was extracted from 200 ng of total RNA using NEXpro™ qRT-PCR Kit. Gene expression analysis by Quantitative PCR has been used for *VEGF* (F: 5’-CCCACTGAGGAGTCCAACAT-3′ and R: 5′-AAATGCTTTCTCCGCTCTGA-3′), and GAPDH (F: 5′-GCATCCTGGGCTACACTGAG-3′ and R: 5′-TCCACCACCCTGTTGCTGTA-3′) used as internal control. Conditions for amplification consisted of an initial denaturing phase at 95 °C for 10 min, proceeded by 40 cycles at 95 °C for 20 secs, at 56 °C for 40 secs, and 72 °C for 60 secs. Extension done at 72 °C for 5 min. The qPCR amplified samples were analyzed by the BiONEER Exicycle™ 96 (BioNEER, Daejeon, South Korea). The Livak method was used to measure fold change between the three groups [15].

### Statistical analysis

The Kruskal–Wallis test was used to compare VEGF expressions in control, distal and proximal hypospadias' tissues. Post hoc analysis used Mann–Whitney U test for comparison of each group. Values were considered statistically significant with *p* < 0.05.

## Results

The age mean was 6.24 ± 4.36 years. Our study consisted of 24 (32%) distal hypospadias, 41 (54.7%) proximal hypospadias, and ten (13.3%) controls. Furthermore, five (6.7%) patients had penoscrotal transposition, and 70 (93.3%) patients were without penoscrotal transposition. Penile curvature in hypospadias was grouped into four groups: mild (< 30 degrees), moderate (30–60 degree), severe (> 60 degrees), and normal with each group number respectively: 19 (25.3%), 15 (20%), 23 (30.7%) and 18 patients (24%) (Table 1).

**Table 1:** Clinical characteristics of patients in our institution

Variable	
All groups (n = 75)	
Age, yr. # mean	6.24 ± 4.36
VEGF, Median (min–max) *	− 0.9 (− 0.1–(− 2.3)
Hypospadias Patients (n = 65)	
Hypospadias Type, n (%)	
*Distal*	24 (32)
Glandular	5 (20.8)
Subcoronal	5 (20.8)
Midshaft	14 (58.4)
*Proximal*	41 (54.7)
Penoscrotal	27 (65.)
Scrotal	13 (31.7)
Perineal	1 (2.4)
*Penile curvature, n (%)*	
Mild (< 30 degree)	12 (16)
Moderate (30–60 degree)	15 (20)
Severe (> 60 degree)	38 (50.7)
Normal	10 (13.3)
*Penoscrotal transposition*	
Yes	7 (9.3)
No	68 (90.7)
Bifid Scrotum	
Yes	7 (9.3)
No	68 (90.7)

VEGF, vascular endothelial growth factor

*Kolmogorov–Smirnov *p* > 0.05

^#^Kolmogorov–Smirnov *p* > 0.05

The qPCR analyses showed decreasing of *VEGF* expression in both distal and proximal (− 1.05 (− 0.1–(− 1.9)); − 1 (− 0.2–(− 2.3))) hypospadias compared with the control group − 0.8 (− 0.4–(− 1.1)), however were not significantly different. The fold change of VEGF expression in proximal and distal hypospadias and controls was not significantly different (mean fold change: hypospadias distal − 0.25; mean fold change: hypospadias proximal − 0.2; *p* = 0.664). Meanwhile, fold change expressions of *VEGF*-related chordees were scaled as follows: (mild − 0.1; moderate 0.1; severe − 0.25; *p* = 0.660) (Table 2).

**Table 2:** Analysis based on hypospadias type and penile curvature severity

Variable	VEGF^#^	Fold change	*p*
*Hypospadias type*			
Distal hypospadias	− 1.05 (− 0.1–(− 1.9))	3.07	0.664
Proximal hypospadias	− 1 (− 0.2–(− 2.3))	2.96	
Control	− 0.8 (− 0.4–(− 1.1))		
*Penile curvature severity*			
Mild	− 0.9 (− 0.2–(− 1.4))	4.60	0.660
Moderate	− 0.7(− 0.1–(− 1.6))	0.723	
Severe	− 1.05 (− 0.1–(− 2.3))	2.03	

VEGF, vascular endothelial growth factor

^#^Kruskal–Wallis test;

## Discussion

In almost every organ, the biomechanical properties of connective tissues perform essential physiological roles. Biochemical and biophysical properties of ECM are responsible for migration, adhesion, the integrity of individual cells, nutrition and differentiation of cells, angiogenesis, and intracellular contact formation [16]. ECM consists of collagenous (different types of collagen) and non-collagenous proteins, such as fibronectin, elastin, laminin, and other components. Elastin, collagen, and proteoglycans are essential factors in the mechanical properties of tissues [17].

Collagen remains possibly the essential component responsible for preserving the structural stability of the tissue. However, the viscoelasticity of biological tissue does not only involve the amount of components' biomechanical properties. It is the product of a dynamic relationship between cells, angiogenic cytokines, and the ECM, such as *VEGF*, which maintains the elasticity and integrity of normal tissues [17]. *VEGF* is a family of closely related growth factors, including several splice variants with numerous biological effects [18]. The production of particular cytokines, including fibroblast growth factor 2 (FGF-2) and *VEGF*, facilitate cell migration and neovascularization in freshly formed scar tissue [19].

Several studies have proved inelastic dartos tissue in hypospadias. The abnormal findings were found in smooth muscle fiber [20], collagen I and VI [4], total collagen, elastin, and reticulin [6]. Even though dartos tissue has abnormal development of some ECM structure, this study found that dartos tissue has well vascularization.

The present study showed a similar expression of *VEGF* in patients with hypospadias compared to the control group. *VEGF* is significantly correlated with the tissue viscoelasticity and the elastic shortening in some organs, such as the cervix. It does not work alone in the elastic mechanism because of crucial tissue mechanical properties such as collagen, elastin, and decorin [9]. A study shows similar blood vessels in the dartos fascia in patients with hypospadias and normal penis [6]. There is strong evidence that this family plays a fundamental role in the growth and differentiation of vascular and lymphatic endothelial cells, but the mechanism remains unclear [9, 18].

*VEGF* is an angiogenic peptide made from macrophages, endothelial cells, and many other cells [21]. It mediates angiogenesis and facilitates the survival of endothelial cells [10]. Integrin β1 acts as a signal transducer to regulate the mechanical environment during this process. Integrin consists of two subunits of transmembrane that modify conformational structures in response to extracellular force [22]. Previous studies identified integrin as a major mechanoreceptor for extracellular signal sensing [23]. Mechanical stimulation enables integrin subunits to be nucleated and clustered to support the maturation of focal adhesions, such as FAK phosphorylation [24].

Pressure overload, particularly integrin β1, can upregulate and activate integrins [25–27]. Interestingly, FAK activation was dispensable in angiogenic response mediated by the elastic modulus [9]. Instead, an elevated elastic module upregulated integrin β1 expression in H9c2 cells and activated its Talin-dependent downstream targets, Akt and PI3K. Activating PI3K/Akt signaling resulted in *VEGF* being upregulated by its primary transcriptional regulator, HIF-1α [28]. Hypoxia-inducible factor-1 (HIF-1) is a primary transcriptional mediator of the O2 homeostasis hypoxic response. Its master regulator consists of two separate subunits, α, and β [29]. HIF-1 (hypoxia-inducible factor-1) is a key transcriptional mediator of the response to hypoxic conditions [30]. Hypoxia is the primary stimulus for VEGF release [31]. Hypoxia also reduces the power of the tissue and predisposes it for rupture [9]. Increased *VEGF* and paracrine are affected by the elevated ECM stiffness in cardiomyocytes. It subsequently stimulates cardiac angiogenesis and the development of *VEGF*, which can be modulated during cardiac hypertrophy through the elastic modulus of the ECM [8]. Accordingly, Wingate also reported that mesenchymal stem cells in soft *VEGF* matrices display more mature endothelial cell markers than MSC on soft non-*VEGF* matrices [32].

However, this study has several limitations, including small sample size and unequal control allocation, a heterogeneous study cohort with both proximal and distal hypospadias. In addition, the tissue studied is the periurethral dartos collected during the excision of the chordee. Most of the distal hypospadias do not require chordee correction or excision. Moreover, VEGF expression needs to be checked in other tissues before a definite conclusion is drawn.

## Conclusions

*VEGF* expressions might not affect the severity of hypospadias and chordee, implying the pathogenesis is complex involving many growth factors. Further study with a larger sample size is necessary to clarify and confirm our findings.

## Data Availability

All data generated or analyzed during this study are included in this published article. The datasets used and/or analyzed during the current study are available from the corresponding author on reasonable request.

## References

[1] Kalfa N, Sultan C (2010). Hypospadias: etiology and current research. Urol Clin N Am.

[2] Yuri P, Heriyanto DS, Rodjani A, Hutasoit YI, Hutahaean AYA, Rahman MR (2020). expression of mrna mastermind-like domain-containing 1, androgen receptor, and estrogen receptor in patients with hypospadias. Open Access Maced J Med Sci.

[3] Zhuang L, Bai M, Zhou W, Yu Y, Wang J (2014). MAMLD1 gene mutation in the incidence of hypospadias in the Chinese population. J Med Biochem.

[4] Yuri P, Gunadi, Lestari RP, Fardilla FP, Setyaningsih WAW, Arfian N (2020). The impact of COL1A1 and COL6A1 expression on hypospadias and penile curvature severity. BMC Urol.

[5] Makovey I, Higuchi TT, Montague DK, Angermeier KW, Wood HM (2012). Congenital penile curvature: update and management. Curr Urol Rep.

[6] Atmoko W, Shalmont G, Situmorang GR, Wahyudi I, Tanurahardja B, Rodjani A (2018). Abnormal dartos fascia in buried penis and hypospadias: evidence from histopathology. J Pediatr Urol.

[7] Devine CJ, Horton CE (1973). Chordee without hypospadias. J Urol.

[8] Shen J, Xie Y, Liu Z, Zhang S, Wang Y, Jia L (2018). Increased myocardial stiffness activates cardiac microvascular endothelial cell via VEGF paracrine signaling in cardiac hypertrophy. J Mol Cell Cardiol.

[9] Sfakianaki AK, Buhimschi IA, Ravishankar V, Bahtiyar MO, Dulay AT, Buhimschi CS (2008). Relationships of maternal serum levels of vascular endothelial growth factor and tensile strength properties of the cervix in a rat model of chronic hypoxia. Am J Obs Gynecol.

[10] Leung DW, Cachianes G, Kuang W, Goeddel DV, Ferrara N (1989). Vascular endothelial growth factor is a secreted angiogenic mitogen. Science.

[11] Senger DR, Claffey KP, Benes JE, Perruzi CA, Sergiou AP, Detmar M (1997). Angiogenesis promoted by vascular endothelial growth factor: regulation through α1 β1 and α2 β1 integrins. Proc Natl Acad Sci.

[12] Senger DR, Ledbetter SR, Claffey KP, Papadopoulos-sergiou A (1996). Stimulation of endothelial cell migration by vascular permeability factor / vascular endothelial growth factor through cooperative mechanisms involving the a bf3 integrin. Am J Pathol.

[13] Wang S, Li X, Parra M, Verdin E, Bassel-duby R, Olson EN (2008). Control of endothelial cell proliferation and migration by VEGF signaling to histone deacetylase 7. Proc Natl Acad Sci.

[14] Lee S, Jilani SM, Nikolova GV, Carpizo D, Iruela-arispe ML (2005). Processing of VEGF-A by matrix metalloproteinases regulates bioavailability and vascular patterning in tumors. J Cell Biol.

[15] Livak KJ, Schmittgen TD (2001). Analysis of relative gene expression data using real-time quantitative PCR and the 2- CT method. Methods.

[16] Koláčná L, Bakešová J, Varga F, Áková E, Plánka L, Necas A (2007). Biochemical and biophysical aspects of collagen nanostructure in the extracellular matrix. Physiol Res.

[17] Gelse K, Po E, Aigner T (2003). Collagens-structure, function, and biosynthesis. Adv Drug Deliv Rev.

[18] Ferrara N (2001). Role of vascular endothelial growth factor in regulation of physiological angiogenesis. Am J Physiol Cell Physiol.

[19] Stroncek JD, Reichert WM. Overview of wound healing in different tissue types. In: Indwelling neural implants. CRC Press, Boca Raton; 2008. 1–37 p. 21204404

[20] Spinoit AF, Van Praet C, Groen LA, Van Laecke E, Praet M, Hoebeke P (2015). Congenital penile pathology is associated with abnormal development of the dartos muscle: a prospective study of primary penile surgery at a tertiary referral center. J Urol.

[21] Ferrara N (2000). VEGF: an update on biological and therapeutic aspects napoleone ferrara. Curr Opin Biotechnol.

[22] Jin M, Andricioaei I, Springer TA (2004). Conversion between three conformational states of integrin i domains with a C-terminal pull spring studied with molecular dynamics. Structure.

[23] Puig M, Lugo R, Gabasa M, Alicia G, Velasques A (2015). Matrix stiffening and beta1 integrin drive subtype-specific fibroblast accumulation in lung cancer. Mol Cancer Res.

[24] Butcher DT, Alliston T, Weaver VM (2009). A tense situation : forcing tumour progression. Nat Rev Cancer.

[25] Fan D, Takawale A, Shen M, Samokhvalov V, Basu R, Patel V (2016). A disintegrin and metalloprotease-17 regulates pressure overload-induced myocardial hypertrophy and dysfunction through proteolytic processing of integrin β 1. Hypertention.

[26] Israeli-rosenberg S, Manso AM, Okada H, Ross RS, Israeli-rosenberg S, Manso AM (2014). Integrins and integrin-associated proteins in the cardiac myocyte. Circ REs.

[27] Li R, Wu Y, Manso AM, Huang MS, Dalton ND, Peterson KL (2012). β1 integrin gene excision in the adult murine cardiac myocyte causes defective mechanical and signaling responses. AM J Pathol.

[28] Lee JS, Yi J-K, An SY, Heo JS (2014). Increased osteogenic differentiation of periodontal ligament stem cells on polydopamine film occurs via activation of integrin and PI3K signaling pathways. Cell Physiol Biochem.

[29] Bardos JI, Ashcroft M (2005). Negative and positive regulation of HIF-1: a complex network. Biochim Biophys Acta.

[30] Zimna A, Kurpisz M (2015). Hypoxia-inducible factor-1 in physiological and pathophysiological angiogenesis : applications and therapies. Biomed Res Int.

[31] Barrientos S, Stojadinovic O, Golinko MS, Brem H, Tomic-canic M (2008). Growth factors and cytokines in wound healing. Wound Repair Regen.

[32] Wingate K, Floren M, Tan Y, Ou P, Tseng N, Tan W (2014). Synergism of matrix stiffness and vascular endothelial growth factor on mesenchymal stem cells for vascular endothelial regeneration. Tissue Eng Part A.

